# Supplementary material for: Demography and productivity during the recovery time sequence of a wild edible bamboo after large-scale anthropogenic disturbance
Source: PLoS One. 2020 Dec 1;15(12):e0243089. doi: 10.1371/journal.pone.0243089 (PMC7707573; doi:10.1371/journal.pone.0243089)
Supplement: S1 Fig — (a) Disturbed areas in each study site in the Teshio Experimental Forest of Hokkaido University. Solid zones and lines in the above map indicate site locations and woodland paths, respectively. Numbers on the above map indicate the site ID numbers. (b-e) Photos of disturbed areas in (b) study site 1, (c) study site 5, (d) study site 8 and (e) study site 11. Photos were taken in May 2014, which is 2 years before the main survey. (DOCX) [file pone.0243089.s001.docx]

**Supporting information to the paper in *PLoS ONE***

Demography and productivity during the recovery time-sequence of a wild edible bamboo after large-scale anthropogenic disturbance

Katayama, N. (n-kata@res.otaru-uc.ac.jp) General Education, Faculty of Commerce, Otaru University of Commerce

**S1 Fig. Map and photos of disturbed areas in each study site in the Teshio Experimental Forest of Hokkaido University.** (a) Disturbed areas in each study site in the Teshio Experimental Forest of Hokkaido University. Solid zones and lines in the above map indicate site locations and woodland paths, respectively. Numbers on the above map indicate the site ID numbers. (b-e) Photos of the disturbed areas in (b) study site 1, (c) study site 5, (d) study site 8 and (e) study site 11. Photos were taken in May 2014, which is 2 years before the main survey.
