# Supplementary material for: Demography and productivity during the recovery time sequence of a wild edible bamboo after large-scale anthropogenic disturbance
Source: PLoS One. 2020 Dec 1;15(12):e0243089. doi: 10.1371/journal.pone.0243089 (PMC7707573; doi:10.1371/journal.pone.0243089)
Supplement: S2 Fig — Blue and red circles indicate the mean value per plot in control and scarification treatments, respectively. Dotted blue lines (P > 0.05) and solid red lines (P < 0.05) denote linear regression in control and scarification treatments, respectively. (DOCX) [file pone.0243089.s002.docx]

**Supporting information to the paper in *PLoS ONE***

Demography and productivity during the recovery time-sequence of a wild edible bamboo after large-scale anthropogenic disturbance

Katayama, N. (n-kata@res.otaru-uc.ac.jp) General Education, Faculty of Commerce, Otaru University of Commerce

**S2 Fig.** **Changes in the number and diameter of old and young bamboo culms after soil scarification**. Blue and red circles indicate the mean value per plot in control and scarification treatments, respectively. Dotted blue lines (*P* > 0.05) and solid red lines (*P* < 0.05) denote linear regression in control and scarification treatments, respectively.
