# Supplementary material for: Demography and productivity during the recovery time sequence of a wild edible bamboo after large-scale anthropogenic disturbance
Source: PLoS One. 2020 Dec 1;15(12):e0243089. doi: 10.1371/journal.pone.0243089 (PMC7707573; doi:10.1371/journal.pone.0243089)
Supplement: S1 Table — (DOCX) [file pone.0243089.s003.docx]

**Supporting information to the paper in *PLoS ONE***

Demography and productivity during the recovery time-sequence of a wild edible bamboo after large-scale anthropogenic disturbance

Katayama, N. (n-kata@res.otaru-uc.ac.jp) General Education, Faculty of Commerce, Otaru University of Commerce

**S1 Table. Number of survey plots at each site.**

| Site ID | Number of survey plots | | Year when soil-scarification was conducted | Years after  disturbance |
| --- | --- | --- | --- | --- |
|  | Disturbance area | Control area |  |  |
| 1 | 4 | 4 | 1972 | 44 |
| 2 | 4 | 4 | 1972 | 44 |
| 3 | 4 | 4 | 1981 | 35 |
| 4 | 4 | 4 | 1985 | 31 |
| 5 | 4 | 4 | 1986 | 30 |
| 6 | 4 | 4 | 1990 | 26 |
| 7 | 4 | 4 | 1992 | 24 |
| 8 | 4 | 4 | 2002 | 14 |
| 9 | 4 | 4 | 2005 | 11 |
| 10 | 10 | 4 | 2010 | 6 |
| 11 | 10 | 4 | 2011 | 5 |
| 12 | 10 | 4 | 2012 | 4 |
| 13 | 10 | 4 | 2013 | 3 |
| 14 | 10 | 4 | 2014 | 2 |
