# Supplementary material for: Demography and productivity during the recovery time sequence of a wild edible bamboo after large-scale anthropogenic disturbance
Source: PLoS One. 2020 Dec 1;15(12):e0243089. doi: 10.1371/journal.pone.0243089 (PMC7707573; doi:10.1371/journal.pone.0243089)
Supplement: S2 Table — (DOCX) [file pone.0243089.s004.docx]

**Supporting information to the paper in *PLoS ONE***

Demography and productivity during the recovery time-sequence of a wild edible bamboo after large-scale anthropogenic disturbance

Katayama, N. (n-kata@res.otaru-uc.ac.jp) General Education, Faculty of Commerce, Otaru University of Commerce

**S2 Table.** **Statistical results of general linear mixed models (GLMMs) about the effects of soil scarification on old and young culms.**

| Dependent variables | Fix factors | *df* | *F* | *P* |
| --- | --- | --- | --- | --- |
| Number of old culms | Scarification | 1, 11.8 | 5.13 | 0.043 |
|  | Year | 1, 132 | 169.78 | <0.001 |
|  | Scarification × Year | 1, 132 | 111.50 | <0.001 |
|  |  |  |  |  |
| Number of young culms | Scarification | 1, 14.6 | 16.33 | 0.001 |
|  | Year | 1, 134 | 114.31 | <0.001 |
|  | Scarification × Year | 1, 134 | 23.76 | <0.001 |
|  |  |  |  |  |
| Diameter of old culms | Scarification | 1, 12.8 | 8.80 | 0.011 |
|  | Year | 1, 76.5 | 10.88 | 0.002 |
|  | Scarification × Year | 1, 77.6 | 23.45 | <0.001 |
|  |  |  |  |  |
| Diameter of young culms | Scarification | 1, 12.0 | 14.51 | 0.003 |
|  | Year | 1, 82.1 | 41.75 | <0.001 |
|  | Scarification × Year | 1, 82.3 | 100.29 | <0.001 |
