# Supplementary material for: Demography and productivity during the recovery time sequence of a wild edible bamboo after large-scale anthropogenic disturbance
Source: PLoS One. 2020 Dec 1;15(12):e0243089. doi: 10.1371/journal.pone.0243089 (PMC7707573; doi:10.1371/journal.pone.0243089)
Supplement: S3 Table — (DOCX) [file pone.0243089.s005.docx]

**Supporting information to the paper in *PLoS ONE***

Demography and productivity during the recovery time-sequence of a wild edible bamboo after large-scale anthropogenic disturbance

Katayama, N. (n-kata@res.otaru-uc.ac.jp) General Education, Faculty of Commerce, Otaru University of Commerce

**S3 Table.** **Statistical results of subsequent general linear mixed models (GLMMs) about the changes in status of old and young culms.**

| Plot | Dependent variables | *df* | *F* | *P* |
| --- | --- | --- | --- | --- |
| Control | Number of old culms | 1, 12.0 | 2.78 | 0.121 |
|  | Number of young culms | 1, 12.1 | 0.05 | 0.832 |
|  | Diameter of old culms | 1, 12.0 | 0.35 | 0.568 |
|  | Diameter of young culms | 1, 12.0 | 0.28 | 0.609 |
|  |  |  |  |  |
| Disturbed | Number of old culms | 1, 11.2 | 33.63 | <0.001 |
|  | Number of young culms | 1, 41.2 | 150.77 | <0.001 |
|  | Diameter of old culms | 1, 11.9 | 42.45 | <0.001 |
|  | Diameter of young culms | 1, 9.57 | 11.75 | 0.007 |
