# Supplementary material for: Demography and productivity during the recovery time sequence of a wild edible bamboo after large-scale anthropogenic disturbance
Source: PLoS One. 2020 Dec 1;15(12):e0243089. doi: 10.1371/journal.pone.0243089 (PMC7707573; doi:10.1371/journal.pone.0243089)
Supplement: Attachment — Submitted filename: Response_to_Reviewers.docx [file pone.0243089.s008.docx]

[Editor]

The authors have revised the manuscript according to the comments of two reviewers. The manuscript has been greatly improved. However one reviewer still has a concern about the English language of this manuscript. I agree with the reviewer and therefore recommend the manuscript be formally accepted after language polishment. A certificate of English editing by a professional company will be greatly appreciated.

[Reviewer #2]

[Comment]

In this version, all comments of the original were addressed soundly. Therefore, the manuscript is recommended to be published with a language polishment.

[Response]

We are very glad to hear these comments, and we apologize for the poor English. The English in this manuscript was carefully checked again by a science editor before the resubmission of this revision. I hope that the revised manuscript is now acceptable for publication in *PLOS ONE.*

Thank you again in advance for your kind consideration of this manuscript.

On behalf of the authors,

Noboru Katayama
